# Role of FDG-PET/CT for monitoring soft tissue tumors

**DOI:** 10.3892/ol.2014.1876

**Published:** 2014-02-12

**Authors:** MANABU HOSHI, NAOTO OEBISU, JUN TAKADA, MAKOTO IEGUCHI, KENICHI WAKASA, HIROAKI NAKAMURA

**Affiliations:** 1 Department of Orthopedic Surgery, Osaka City University Graduate School of Medicine, Osaka 545-8585, Japan; 2 Department of Diagnostic Pathology, Osaka City University Graduate School of Medicine, Osaka 545-8585, Japan

**Keywords:** soft tissue tumor, 2-deoxy-2-F^18^-fluoro-D-glucose positron emission tomography, computed tomography, maximum standardized uptake value, prognosis, metastasis, recurrence

## Abstract

The aim of the current study was to evaluate the limitations of 2-deoxy-2-F^18^-fluoro-D-glucose positron emission tomography combined with computed tomography (FDG-PET/CT) when monitoring soft tissue tumors. The diagnostic criteria of malignancy was defined as the tumor having a maximum standardized uptake value (SUVmax) ≥2.0 and a maximum diameter ≥5 cm as measured using FDG-PET/CT. One-hundred-and-thirteen patients, that were either included in the criteria or not, were compared. In addition, the values of SUVmax of the primary tumor and relapse in 12 patients were evaluated. The Kaplan-Meier analysis demonstrated that patients with tumors measuring ≥5 cm size and ≥2.0 SUVmax were associated with a worse survival rate. Among the 12 patients with relapse, statistical significances were detected in the tumor diameters, however, not in the SUVmax values. Thus, the criteria identified patients that were associated with a poor prognosis, and the SUVmax of distant metastases and local recurrences were identified to be significantly affected by tumor size.

## Introduction

Integrated 2-deoxy-2-F^18^-fluoro-D-glucose positron emission tomography combined with computed tomography (FDG-PET/CT) has increasingly been used for the management of patients with various types of cancer, including soft tissue tumors. FDG-PET can determine the tumor characteristics of high metabolism and an increased rate of glucose utilization compared with normal tissues. The high capacity of glucose utilization is a possible reflection of the malignant nature of a tumor. A high maximum standardized uptake value (SUVmax) is one of the informative biomarkers measured using this modality for the differential diagnosis between malignant and benign tumors. Numerous studies (1–3) previously proposed that a threshold SUVmax value of 1.9–2.0 may contribute to the differential diagnosis. Conversely, CT can evaluate the size of soft tissue tumors, and it is well-known that a tumor size ≥5 cm is highly likely to be a malignant soft tissue tumor (4,5). Integrated FDG-PET/CT, which can evaluate tumor function and perform a morphological assessment at the same time, may therefore have an advantage for differentiating between a malignant and a benign soft tissue tumor.

Certain malignant soft tissue tumors frequently develop to distant metastases and local recurrences. Integrated FDG-PET/CT is able to rapidly predict the biological activity of tumors (6,7). SUVmax values in integrated FDG-PET can predict biological activity of tumors, as well as the tumor grade. SUVmax values of metastatic and recurrent tumors are generally considered to be higher when compared with primary lesions (8); however, few studies have focused on this issue in the field of soft tissue tumors.

To monitor soft tissue tumors at follow-up, the orthopedic oncologists and radiologists must be aware of the capabilities and limitations of integrated FDG-PET/CT for the evaluation of soft tissue tumors. The present study was composed of two clinical studies. First, the diagnostic criteria for malignant soft tissue tumors were defined as a tumor ≥5 cm in size and an SUVmax of ≥2.0; this was interpreted using integrated FDG-PET/CT. Furthermore, the efficacy of these criteria in differentiating malignant from benign soft tissue tumors and establishing patient prognoses was examined. Second, the role of integrated FDG-PET/CT in comparing metastases/recurrences to primary tumors in the same individuals was investigated.

## Materials and methods

From our database comprising of 243 bone and soft tissue tumors, which were obtained from patients who were examined by pre-operative integrated FDG-PET imaging during the period from December 2004 to December 2012, 113 patients with soft tissue tumors were biopsied or surgically treated, pathologically recognized, and followed up at the Department of Orthopedic Surgery, Osaka City University Graduate School of Medicine (Osaka, Japan). These patients consisted of 58 males and 55 females, ranging in age from 17 to 91 years (mean age, 56.2±16.6 years). All of the follow-up patient data were available and the median follow-up period was 24.5±20.9 months. Clinical information was retrospectively reviewed in the present study and focused on clinical features, radiological findings, histopathology and prognoses of the patients, compared with SUVmax.

In accordance with previous studies (1–5), the diagnostic criteria for a malignant soft tissue tumor using integrated FDG-PET/CT was defined as a tumor sized ≥5 cm with an SUVmax ≥2.0. The sensitivity and accuracy was calculated using these criteria and the patient survival curve was estimated using the Kaplan-Meier method. Group 1 (G1) is formed of patients with a tumor size <5 cm and/or SUVmax <2.0. Group 2 (G2) is formed of patients with a tumor size ≥5 cm and SUVmax ≥2.0.

Distant metastasis and local recurrence were newly identified using integrated FDG-PET/CT in a total of 12 patients between the initial visit and the last follow-up. These patients consisted of nine males and three females, ranging in age from 22 to 83 years (56.6±18.1 years). The values of SUVmax were measured in primary and metastatic/recurrent lesions in the same patients. The metastases or local recurrence following identification using integrated FDG-PET/CT was determined through histological diagnosis on surgically resected materials, radiological magnetic resonance imaging (MRI) or CT. In addition, the clinical information of the patients was retrospectively reviewed in the present study. All follow-up patient data were available.

The study protocol was approved by the institutional ethics review board of Osaka City University Graduate School of Medicine.

### FDG-PET/CT scanning

All patients had previously undergone routine evaluation by plain radiography and CT and/or MRI at Osaka City University Hospital (Osaka, Japan) or at the referring institution. Patients fasted for ≥4 h prior to the FDG-PET study to standardize the imaging conditions. To avoid data contamination, patients with blood glucose levels >150 mg/dl were excluded from the study. CT and PET images were routinely acquired from the orbit to the proximal thigh 60 min after intravenous injection of 2.7 MBq/kg of FDG. If necessary, additional images were captured of the toes. PET was performed using a whole body PET/CT scanner (Discovery ST; GE Healthcare, Tokyo, Japan). For the CT scan portion of the study, the settings were as follows: 140 kVp; 50 mA (Auto mA); pitch, 1.75; slice thickness, 3.27 mm; beam collimation, 20 mm; field of view (FOV), 500 mm; and matrix size, 512×512, with breathing at rest. For the PET portion of the study, a 3-dimensional acquisition was performed; slice thickness, 3.27 mm; reconstruction interval, 3.27 mm; FOV, 500 mm; and matrix size, 128×128, using the Ordered Subsets Expectation Maximization Reconstruction method, with 17-mm overlap and a Gaussian filter (9).

The FDG-PET/CT images were analyzed by a radiologist who was unaware of the histology and all of the FDG-PET/CT studies were analyzed quantitatively. The SUVmax was measured within the axial image slice with the highest concentration of FDG activity. The SUV was defined as follows: SUV = radioactivity concentration in tissue (Bq/g) / [injected dose (Bq)/patient’s body weight (g)]. The regions of interest were determined using the pixel with the highest FDG accumulation (SUVmax).

### Histological examination

Biopsies or surgically resected specimens were fixed in 10% formalin and routinely processed for paraffin embedding. The sections were cut to a 4-μm thickness and stained with hematoxylin and eosin and the final diagnoses of the lesions were histologically determined. All biopsies and resected specimens were assessed by pathologists with specific training and expertise in bone and soft tissue tumors; the investigators were blinded to the findings of the FDG-PET/CT studies. The diagnoses followed the World Health Organization classification system (10).

### Statistical analysis

Quantitative data are presented as the mean ± standard deviation, median and range. The Mann-Whitney U test and Kruskal-Wallis one-way analysis of variance were used for unpaired comparisons between the quantitative parameters. Patient survival was estimated using the Kaplan-Meier survival method between the patients diagnosed with malignant and non-malignant tumors. The relevant time scale was analyzed from the time of the FDG-PET/CT study to the last follow-up and log-rank tests were used to evaluate the differences. Statistical analysis was performed using Excel statistics software (version 2012; SSRI Co., Ltd.) for Windows. P<0.05 was considered to indicate a statistically significant difference.

## Results

### Details of each histological tumor type and SUVmax

A total of 113 patients with soft tissue tumors were included in the present study. The SUVmax for each histological subtype is summarized in Table I. The majority of malignant tumors demonstrated a high SUVmax, while well-differentiated liposarcoma, low-grade myofibroblastic sarcoma, myxoinflammatory fibroblastic sarcoma, malignant mixed tumor and extraskeletal chondrosarcoma demonstrated a low SUVmax of <2.0. In benign tumors, schwannoma, neurofibroma, desmoid, hematoma and sarcoidosis demonstrated a relatively high SUVmax of ≥2.0.

### Clinical information of soft tissue tumors and SUVmax

The final diagnosis revealed 19 benign lesions and 94 malignant bone tumors. There was a statistically significant difference identified in the SUVmax between the intensity of the benign (2.9±2.2) and the malignant (4.8±3.9) soft tissue tumors (P=0.01); however, no statistically significant differences were observed when comparing primary and metastatic tumors, age (older or younger than 60 years old), tumor site (extremity or trunk), tumor size at the greatest diameter (<5, 5–10 or >10 cm) and depth (superficial or deep).

### Tumor size and SUVmax on PET/CT findings in comparison with histology

**Figure 1:**
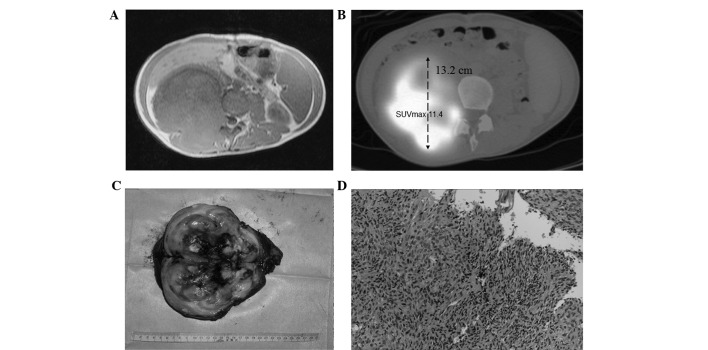
Malignant peripheral nerve sheath tumor of the right retroperitoneum in an 18-year-old male with neurofibromatosis 1. (A) T1-weighted images by magnetic resonance imaging showed an isointense mass. (B) 2-deoxy-2-F^18^-fluoro-D-glucose positron emission tomography combined with computed tomography demonstrated a tumor of 13.2 cm at the largest diameter and an SUVmax of 11.4. (C) Resected specimen confirmed the myxoid tumor with necrosis. (D) Microscopically, spindle cells were observed. The patient was diagnosed with a malignant peripheral nerve sheath tumor (H&E staining; magnification, ×200). SUVmax, maximum standardized uptake value.

The sensitivity and accuracy were calculated using the criteria described in the present study for malignancy of tumors size ≥5 cm and an SUVmax ≥2.0, in comparison with the histological results (Fig. 1). Sensitivity and accuracy were calculated to be 55.3 and 54.0%, respectively (Table II).

### Survival curve of the patients with tumors sized ≥5 cm and an SUVmax ≥2.0

**Figure 2:**
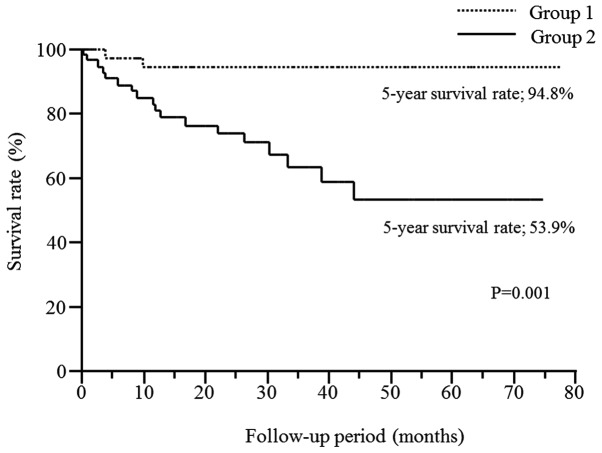
Kaplan-Meier analysis between patients with soft tissue tumors. Group 1 (G1) represents the survival curve of patients with a tumor size <5 cm and/or SUVmax <2.0. Group 2 (G2) represents patients with a tumor size ≥5 cm and SUVmax ≥2.0. The five-year survival rate was 94.8% in G1, and 53.9% in G2. There was a statistically significant difference between the survival rates (P=0.01).

The Kaplan-Meier analysis demonstrated that the five-year survival rate was 53.9% in patients with tumors sized ≥5 cm and an SUVmax ≥2.0 (G2), while that of the patient excluded from group 1 was 94.8% (G1). The difference was statistically significant (P=0.001; log-rank test) (Fig. 2).

### Clinical information of patients with metastatic/recurrent tumors

A total of 12 patients with soft tissue tumors were included in the present study. The clinical information, anatomical site, histopathology, tumor size and SUVmax of primary and metastatic/recurrent lesions is summarized in Table III. With regard to prognosis, eight patients died of disease (DOD), three were alive with disease (AWD) and one had no evidence of disease (NED).

### Comparison of SUVmax and tumor size on FDG-PET/CT between primary and metastatic/recurrent lesions

The mean SUVmax of primary and metastatic/recurrent tumors were 5.9±3.0 and 6.1±3.4, respectively. No statistical significances were identified between them. The mean values of the greatest diameter of primary tumors and metastasis/recurrence were 7.0±3.1 cm and 3.7±2.2 cm, respectively. The primary tumor was significantly larger than the metastatic/recurrence tumors (P=0.00029).

## Discussion

Kern *et al* (11) first applied FDG-PET to soft tissue tumors, including malignant fibrous histiocytoma; it has since been shown to be one of the most powerful diagnostic tools in oncology, enabling the functional assessment of soft tissue tumors. Currently, FDG-PET can identify the metabolic rate of glycolysis in tumors and is increasingly applied to grading (12,13), staging (14), chemotherapeutic response assessment (15,16) and surgical planning (3) of soft tissue tumors. Preliminary reports emphasized the ability of FDG-PET to distinguish benign from malignant tumors (1–3,17). However, numerous studies (18,19) have raised the question that if FDG-PET cannot differentiate malignant from benign soft tissue tumors in the presence of false positive findings from aggressive benign tumors and inflammatory lesions, then is it an insufficient technique to judge between benign and malignant bone tumors? However, recently FDG-PET/CT analysis has been re-examined and its efficiency was investigated (20). Bischoff *et al* (21) reported that the usefulness of FDG-PET/CT in soft tissue and osseous tumors had a sensitivity of 69–80%, a specificity of 83–100% and an accuracy of 79–86%; although their criteria for interpreting malignant tumors were obscure and required the judgment of radiologists from numerous sources of information for conventional imaging. Charest *et al* (20) demonstrated a high sensitivity for the accurate discrimination between low- and high-grade sarcomas, however, not between benign and malignant soft tissue tumors. We doubt whether FDG-PET/CT is able to accurately differentiate malignant from benign tumors. In the present study, distinct criteria were designed to interpret malignant tumors and the efficacy of establishing a differential diagnosis using these criteria was evaluated.

Previous studies were referred to in order to define the criteria between malignant and benign tumors. In FDG-PET analysis, Feldman *et al* (2) proposed an SUVmax of 2.0 with a high sensitivity of 97.7% and a high specificity of 100%, while Watanabe *et al* (3) calculated the SUVmean of 1.9 with a high sensitivity of 100% and a high specificity of 76.9%. In the present study, an SUVmax of 2.0 was defined as the threshold value in FDG-PET for glucose metabolism in tumors. Furthermore, FDG-PET/CT is able to perform morphological measurements of tumor size at the same time, in addition to the functional assessment. A size of ≥5 cm has been used as an indicator of possibly malignant soft tissue tumor (5). Thus, combining the findings of SUVmax ≥2.0 and tumor size ≥5 cm was expected be a highly sensitive detector of malignant tumor in FDG-PET/CT. The results of the present study indicated that the sensitivity and accuracy, that were based on the proposed criteria, were 55.3 and 54.0%, respectively, for the differential diagnosis between malignant and benign tumors. Contrary to expectations, these data indicate that the criteria set out in the present study were insufficient to enable a differential diagnosis using integrated FDG-PET/CT. Aoki *et al* (19) previously denied the usefulness of a threshold value in distinguishing malignant and benign soft tumors, owing to a false positive overlap by histiocytic, fibroblastic and neurogenic tumors. Inflammatory processes also enhanced FDG uptake (22), although the mechanism remains to be completely understood.

Previously, concerning specific histological subtype, such as osteosarcoma (23), Ewing sarcoma (24) and rhabdomyosarcoma (25), FDG-PET was assessed as a useful modality for prognosis. Metabolic reduction after chemotherapy on FDG-PET may be a useful response marker in high-grade sarcomas (26). According to the Kaplan-Meier analysis in the present study, patients with a tumor size ≥5 cm and an SUVmax ≥2.0 were associated with worse survival, compared with those without these characteristics. The tumors sized ≥5 cm with an SUVmax ≥2.0 may be malignant soft tissue sarcomas. In the present study, false positive tumors that were identified in the benign tumors were schwannoma (n=5), neurofibroma (n=2), desmoid (n=2), hematoma (n=1), sarcoidosis (n=1) and giant cell tumor of the tendon sheath (n=1). The sizes of these benign tumors were generally small (<5 cm) and they were divided into G1. Whereas, the false negative tumors in the malignant tumors were well-differentiated liposarcoma (n=6), low grade myxofibroblastic sarcoma (n=2), myxoinflammatory fibroblastic sarcoma (n=1), malignant mixed tumor (n=1) and extraskeletal chondrosarcoma (n=1). The majority of these negative false tumors were interpreted as potentially low-grade sarcoma, even when the size was >5 cm. These tumors may also be classified as G1. Therefore, the criteria of tumors sized ≥5 cm with an SUVmax ≥2.0 on integrated FDG-PET/CT is likely to be an indicator of a worse prognosis in soft tissue tumors.

Tateishi *et al* (6) and Arush *et al* (7) described that FDG-PET/CT was useful in identifying metastasis of musculoskeletal tumors as a screening of sarcoma and was superior to conventional images. Yanagawa *et al* (8) demonstrated that metastatic bone tumors exhibited a higher SUVmax compared with that of primary tumors. It appears likely that metastatic tumors acquire a more aggressive nature than primary tumors. Additionally, a previous study reported that a higher pathological grade of tumor resulted in a higher SUVmax in FDG-PET/CT (12). In the present study, the SUVmax on FDG-PET/CT for metastatic/recurrent tumor was not higher than that of primary lesions, which was contrary to the prediction that metastatic tumors may have a higher SUV than primary tumors.

Although no significant difference in the value of SUVmax between primary and metastatic/recurrent tumors was detected, these results may be dependent on the tumor size. The size of the primary tumor, estimated as the greatest diameter, was demonstrated to be significantly larger than that of metastatic/recurrent tumor. The periodical follow-ups with FDG-PET/CT contributed to finding the small-size metastatic/recurrent tumors in the present study. In a previous study, there was a correlation between the size of the tumor and sensitivity of the FDG-PET (27). Gould *et al* (28) also published a meta-analysis of 1,474 pulmonary nodules that were evaluated by FDG-PET and concluded that FDG-PET had an overall high specificity (96.8%) but variable sensitivities (77.8%) for nodules <1 cm. Fortes *et al* (27) supported that the small size of lung metastasis from sarcoma was markedly less sensitive than other carcinoma in FDG-PET. The glucose metabolism of sarcoma in FDG-PET/CT should be affected by the size of the tumors (29).

There are several limitations of the present study, including its retrospective nature, the limited number of patients enrolled and the potential selection bias of the patients. The proportion of malignant tumors that were studied was greater than the proportion of benign tumors. The study included numerous types of soft tissue tumors and their histologies were not matched, although soft tissue tumors arise from various points of origin. It would be difficult to isolate cases to an individual origin, due to its rarity. Schwab and Healey (30) demonstrated that FDG-PET may lack the sensitivity for myxoid liposarcoma metastasis, due to the inability to detect glucose utilizing cells within the myxoid matrix. Similarly, certain sarcoma may show a peculiar FDG uptake pattern. Further study was necessary to establish each specific histological subtype for accurate examination in the present study. The size of the tumor is also an important diagnostic problem that is related to the capability of FDG-PET/CT in detecting tumors (31). The smallest diameter of the metastatic tumor in the present study was 1.1 cm; a previous study identified that a lesion of size 5 mm could not be evaluated adequately by FDG-PET (32). A prospective study is required to overcome these limitations and confirm the results of the present study.

In conclusion, the diagnostic criteria of tumor size ≥5 cm and SUVmax ≥2.0 on integrated FDG-PET/CT was insufficient for distinguishing malignant from benign soft tissue tumors, owing to false positive benign tumors and false negative malignant tumors. However, the Kaplan-Meier analysis demonstrated that patients meeting these criteria were associated with a worse prognosis. The SUVmax on FDG-PET/CT was compared between primary and metastatic/recurrent tumors and no significant difference identified between their values. Although the idea of whole body cancer surveillance using FDG-PET/CT is fascinating as a screening tool for the recurrence of sarcoma, orthopedic oncologists and radiologists must be aware that FDG-PET/CT assessments are limited by the tumor size.

## Figures and Tables

**Table I:** Details of each histological tumor type and SUVmax.

Type (no. of cases)	SUVmax
Malignant	
Myxoid liposarcoma (22)	2.9±2.1
Pleomorphic liposarcoma (21)	6.5±3.2
Leiomyosarcoma (7)	6.5±8.6
Well-differentiated liposarcoma (6)	1.0±0.9
Malignant peripheral nerve sheath tumor (5)	7.9±2.0
Synovial sarcoma (4)	3.6±1.5
Epithelioid sarcoma (4)	6.9±0.7
Dedifferentiated liposarcoma (3)	3.5±1.4
Gastrointestinal mesenchymal tumor (3)	4.8±3.4
Pleomorphic malignant fibrous histiocytoma (3)	7.8±3.8
Low grade myofibroblastic sarcoma (2)	1.5±0.7
Myxofibrosarcoma (2)	2.5±2.5
Solitary fibrous tumor (2)	2.2±0.7
Alveolar soft part sarcoma (1)	9.0
Clear cell sarcoma (1)	3.9
Malignant hemangiopericytoma (1)	13.7
Myxoinflammatory fibroblastic sarcoma (1)	0.9
Malignant mixed tumor (1)	1.3
Extraskeletal osteosarcoma (1)	16.3
Mesothelioma (1)	2.0
Extraskeletal chondrosarcoma (1)	1.9
Extraskeletal myxoid chondrosarcoma (1)	5.6
Extraskeletal mesenchymal chondrosarcoma (1)	6.2
Benign	
Schwannoma (5)	4.1±2.4
Lipoma (3)	0.7±0.4
Neurofibroma (2)	2.4±1.1
Desmoid (2)	3.8±0.5
Nodular fasciitis (2)	1.8±2.5
Hemangioma (1)	1.3
Hematoma (1)	3.0
Ganglion (1)	1.0
Sarcoidosis (1)	5.5
Giant cell tumor of tendon sheath (1)	2.2

Data are presented as the mean ± SD. SUVmax, maximum standardized uptake value.

**Table II:** Tumor size and SUVmax on findings from FDG-PET/CT in comparison with the histology results.

	Malignant	Benign	Total
Tumor size ≥5 cm and SUVmax ≥2.0	52	10	62
Tumor size <5 cm and/or SUVmax <2.0	42	9	51
Total	94	19	113

Sensitivity, specificity, and accuracy were calculated for tumors ≥5 cm and SUVmax ≥2.0. Sensitivity, specificity and accuracy were 55.3, 47.7 and 54.0%, respectively. SUVmax, maximum standardized uptake value.

**Table III:** Clinical information of patients with metastatic/recurrent tumors.

		Primary tumor					Metastasis/recurrence				
					
Case	Age (years)	Gender	Primary site	Histopathology	Size (cm)	SUVmax	Site	Type	Size (cm)	SUVmax	Prognosis
1	22	F	Lower leg	Alveolar soft part sarcoma	5.9	8.9	Lumbar vertebrae	Metastasis	2.8	8.9	DOD
2	27	M	Forearm	Epithelioid sarcoma	3.2	7.2	Lung	Metastasis	1.6	2.2	AWD
3	52	M	Thigh	Pleomorphic liposarcoma	8.5	9.2	Humerus	Metastasis	3.1	2.2	DOD
4	59	M	Forearm	MIFS	5.2	0.9	Buttock	Metastasis	3.7	6.6	DOD
5	75	M	Thigh	Myxoid liposarcoma	3.4	2.5	Thigh	Recurrence	4.8	2.7	AWD
6	71	F	Forearm	Leiomyosarcoma	6.5	2.5	Forearm	Recurrence	1.4	4.8	NED
7	73	M	Thigh	Pleomorphic liposarcoma	11.9	5.5	Pubic bone	Recurrence	9.2	7.7	DOD
8	46	M	Chest wall	Clear cell sarcoma	4.8	3.4	Lung	Metastasis	4	3.9	AWD
9	79	F	Thigh	Pleomorphic liposarcoma	6.2	7.1	Ileocecal	Metastasis	2	9.7	DOD
10	56	M	Shoulder	Pleomorphic malignant fibrous hystiocytoma	10.1	6.7	lung	Metastasis	2	2.7	DOD
11	46	M	Groin	Epithelioid sarcoma	5.4	6.3	Groin	Metastasis	3.8	9.4	DOD
12	83	M	Chest wall	Pleomorphic liposarcoma	12.4	10.3	Liver	Metastasis	5.7	11.8	DOD

SUVmax, maximum standardized uptake value; F, female; M, male; MIFS, myxoinflammatory fibroblastic sarcoma; DOD, died of disease; AWD, alive with disease; NED, no evidence of disease.
